# Effect of Self-Directed Home Therapy Adherence Combined with TheraBracelet on Poststroke Hand Recovery: A Pilot Study

**DOI:** 10.1155/2023/3682898

**Published:** 2023-03-08

**Authors:** Gabrielle Scronce, Viswanathan Ramakrishnan, Amanda A. Vatinno, Na Jin Seo

**Affiliations:** ^1^Department of Health Sciences and Research, College of Health Professions, Medical University of South Carolina, Charleston, SC, USA; ^2^Ralph H. Johnson VA Health Care System, Charleston, SC, USA; ^3^Department of Public Health Sciences, College of Medicine, Medical University of South Carolina, Charleston, SC, USA; ^4^Division of Occupational Therapy, Department of Rehabilitation Sciences, College of Health Professions, Medical University of South Carolina, Charleston, SC, USA

## Abstract

Hand impairment is a common consequence of stroke, resulting in long-term disability and reduced quality of life. Recovery may be augmented through self-directed therapy activities at home, complemented by the use of rehabilitation devices such as peripheral sensory stimulation. The objective of this study was to determine the effect of adherence to self-directed therapy and the use of TheraBracelet (subsensory random-frequency vibratory stimulation) on hand function for stroke survivors. In a double-blind, randomized controlled pilot trial, 12 chronic stroke survivors were assigned to a treatment or control group (*n* = 6/group). All participants were instructed to perform 200 repetitions of therapeutic hand tasks 5 days/week while wearing a wrist-worn device 8 hours/day for 4 weeks. The treatment group received TheraBracelet vibration from the device, while the control group received no vibration. Home task repetition adherence and device wear logs, as well as hand function assessment (Stroke Impact Scale Hand domain), were obtained weekly. Repetition adherence was comparable between groups but varied among participants. Participants wore the device to a greater extent than adhering to completing repetitions. A linear mixed model analysis showed a significant interaction between repetition and group (*p* = 0.01), with greater adherence resulting in greater hand function change for the treatment group (*r* = 0.94; *R*^2^ = 0.88), but not for the control group. Secondary analysis revealed that repetition adherence was greater for those with lower motor capacity and greater self-efficacy at baseline. This pilot study suggests that adherence to self-directed therapy at home combined with subsensory stimulation may affect recovery outcomes in stroke survivors. This trial is registered with NCT04026399.

## 1. Introduction

Stroke is a major medical event that occurs in nearly 800,000 people in the United States each year [1]. Upper extremity (UE) sensorimotor impairment is a common consequence of stroke, affecting 77% of stroke survivors [2]. UE sensorimotor impairment decreases individuals' ability to perform functional activities for self-care, hygiene, employment, and recreation, thereby diminishing their independence and quality of life [3, 4].

Research shows that extensive practice of task-specific activities results in improved functional recovery of the UE poststroke [5–9]. However, the high amount of UE activity necessary for functional recovery [5] cannot be achieved within typical therapy sessions [7, 10–12]. To circumvent the limited time available with a therapist, a home exercise program (HEP) consisting of self-directed therapeutic activity is commonly prescribed as part of the standard therapy [13]. However, adherence to HEP varies substantially among patients [13–16]. Varying adherence levels have been shown to explain the variability in recovery of overall physical mobility post-stroke [17–19]. However, a relationship has not been studied between UE HEP adherence and UE functional outcome.

**Figure 1:**
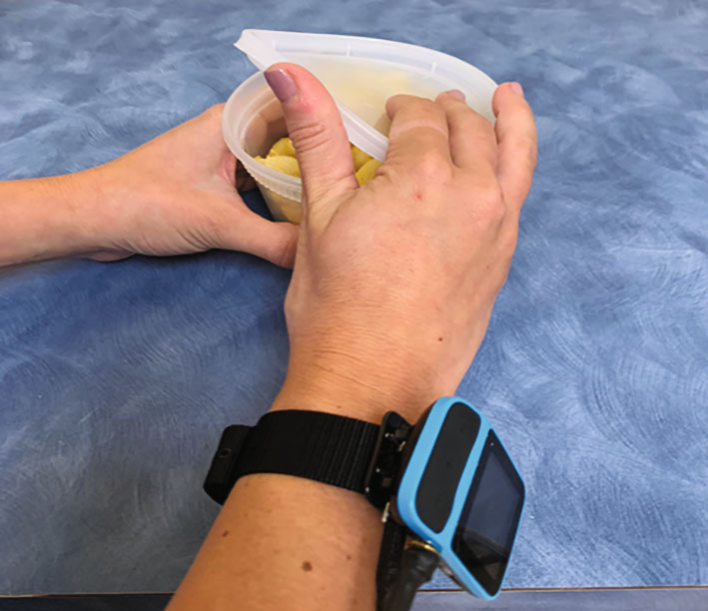
TheraBracelet worn on paretic wrist during task practice.

HEP can be complemented by rehabilitation devices, such as a peripheral sensory stimulation device [20]. Meta-analysis has shown that the use of peripheral sensory stimulation along with UE therapy can the increase functional recovery of the UE [21]. Previously used peripheral sensory stimulation is typically at a suprathreshold level. Suprathreshold stimulation applied before each UE therapy session [21] lengthens the treatment durations and can lower patient adherence [20, 22]. Suprathreshold stimulation applied during therapy sessions can interfere with sensory feedback required for manipulation of objects. Therefore, a new stimulation device named TheraBracelet [23] (Figure 1) has been proposed. TheraBracelet is imperceptible random-frequency vibration applied to the wrist skin [23]. TheraBracelet does not interfere with UE hand tasks since the stimulation is imperceptible and delivered via a small device worn on the wrist like a watch [24–26]. TheraBracelet vibration stimulates mechanoreceptors in the skin and subsequently afferent neurons [27, 28], thereby adding small random currents to neurons in the sensorimotor cortex [29]. These small random currents trigger coherent [30] neuronal firing [29, 31, 32] during the performance of hand tasks and enhance neural communication [33, 34] required for hand tasks [35–39] via stochastic facilitation [35]. As a result, TheraBracelet has demonstrated the potential to improve finger touch sensation [26, 36, 38] and dexterity [23, 37, 40, 41], as well as functional recovery [23, 40].

Preliminary efficacy of TheraBracelet has been examined in the laboratory setting [23, 40]. However, the efficacy of using TheraBracelet in conjunction with HEP in stroke survivors' homes has not been examined. As the logical next step, the objective of this pilot study was to determine the effect of adherence to UE HEP combined with TheraBracelet on hand function for stroke survivors. Our hypothesis was that the combination of greater adherence to UE HEP and receiving TheraBracelet stimulation would result in greater improvement in hand function [42].

## 2. Materials and Methods

### 2.1. Participants

Inclusion criteria were adult (age ≥18 years), chronic stroke survivors (≥6 months post-stroke) with tactile sensory deficits of the fingertips (Monofilament [43] score>2.83, 2-Point Discrimination Test [44] score>5 mm, or sense of numbness based on verbal report). Additional inclusion criteria were the ability to put on a watch daily (with or without caregiver help) and ability to move objects with the paretic hand as necessary to perform HEP. Exclusion criteria included complete upper limb deafferentation, rigidity (Modified Ashworth Scale [45] = 4), UE botulinum toxin injection within 3 months prior to enrollment or during enrollment, brainstem stroke, comorbidity (peripheral neuropathy, orthopedic conditions in the hand that limit motion [46], premorbid neurologic conditions, compromised skin integrity of the hand/wrist unrelated to stroke, such as from long term use of blood thinners), change in neurological disorder medications during enrollment, concurrent upper extremity rehabilitation therapy, and language barrier or cognitive impairment that precluded following instructions or providing consent. All participants signed a consent form that was approved by the Medical University of South Carolina (MUSC) Institutional Review Board before participation in the study.

### 2.2. Study Design

This was a pilot, double-blind, randomized controlled trial in which chronic stroke survivors were randomly assigned to a treatment or control group. All participants were instructed to perform HEP consisting of 200 repetitions of therapeutic UE tasks 5 days/week for 4 weeks. In addition, participants were provided a wearable prototype device for TheraBracelet, which has been shown to be successfully worn by stroke survivors in their homes every day during daily living without safety issues [25]. All participants were instructed to wear the device at least 8 hours/day every day for 4 weeks, consistent with the previous study [25]. While the control group received no vibration from the device, the treatment group received subsensory TheraBracelet vibration from the device. The device, which is further described in Seo et al. [25], provided continuous vibration at 60% of the participant's sensory threshold, determined at each visit. To ensure blinding of researchers, different research personnel administered the device to the participant, who was not the research therapist who administered HEP and assessments.

### 2.3. Home Exercise Program (HEP)

Each week, beginning with the baseline visit, participants met individually with an occupational therapist and were administered HEP with specific tasks to practice at home for the following week. Tasks were selected from a menu of task practice activities that was developed by two experienced occupational therapists based on the EXCITE trial [47] manual and the task-specific training text by Lang and Birkenmeier [48]. Tasks included self-care, household, leisure, and vocational tasks and were separated into two types: tasks requiring [1] primarily in-hand manipulation and [2] reaching. The participant and therapist collaboratively selected 2 tasks of each type that the participant considered meaningful to perform during the week. The selection from a menu ensured consistency while allowing saliency of selected tasks to increase motivation for the participant to complete the task [47]. When possible, tasks were selected to be more challenging than previous weeks' tasks so that the intervention was both individualized and progressive [11, 48]. In addition, the therapist provided written options to grade each task to make it easier or harder at the participant's discretion to allow the participant to be challenged but not overwhelmed by the task for optimal neural plasticity [48]. See Table 1 for task and grading examples.

Participants were instructed to complete each of the 4 selected tasks 50 times per day, 5 days per week so that participants would complete 4000 repetitions of task-specific practice over the 4-week intervention. This dose was selected because it was considered feasible for home-based, self-directed practice within a 1-2 hour timeframe [11, 49, 50] while corresponding to the lower end of repetitions that have been shown to promote neural plasticity and functional recovery in animal and motor learning studies [11, 51, 52].

To facilitate adherence, a transfer package [53] was implemented that included a contract for adherence [54], a written log to track HEP adherence, and problem solving to overcome barriers to completing HEP [55–57]. In the contract, the participant agreed to adhere to the intervention including completing all HEP assignments and using the paretic hand on specific activities of daily living as much as possible outside the lab. The contract was signed by the participant and therapist to emphasize its importance [54]. To track HEP adherence, participants were provided a paper log to record the number of repetitions completed for each of their prescribed HEP tasks for each day. At the weekly meetings, if HEP adherence according to the written log was less than 100%, the therapist facilitated a discussion with the participant to help them think through barriers to completing HEP and ways to overcome them [55–57].

### 2.4. Assessments

At the weekly meetings, HEP adherence, device wear logs, and hand function assessment were obtained. HEP adherence and device wear information was obtained from the paper log in which participants recorded the number of repetitions for each task they completed as well as the time they put on and took off the device for each day. Average percent HEP adherence was defined as the percentage of HEP repetitions completed out of the number prescribed.

Hand function, the primary outcome, was assessed by the Stroke Impact Scale (SIS) Hand domain [58, 59]. The SIS was used because it is a stroke-specific, self-report measure with high test-retest reliability, concurrent validity, and responsiveness to change [59, 60] and because this assessment could be administered by phone during COVID-19 quarantine when in-person visits were restricted.

To characterize the participant pool, demographic information was obtained at baseline. Baseline assessment also included motor function and self-efficacy, as they may affect HEP adherence and functional recovery [61, 62]. In particular, baseline motor capacity was measured by Box and Blocks Test (BBT), a functional performance test of upper limb motor capacity with high validity, test-retest reliability, ability to detect change, and clinical utility [63–65]. The BBT score represents the number of blocks moved in one minute [63] with the affected hand. For self-efficacy, we implemented a 4-item, self-report measure tailored to the language of UE rehabilitation therapy (see appendix) [66–68]. Specifically, participant's knowledge and confidence in taking responsibility for their UE treatment were scored on a Likert scale from 1 (disagree strongly) to 4 (agree strongly).

Adverse events were explicitly asked and recorded at each weekly meeting. To assess maintenance of blinding of participants, a questionnaire was administered at the end of the study. It asked whether participants had felt the device vibrating during the past month, and if they did how long they felt the vibration.

### 2.5. Analysis

Baseline characteristics were summarized using means and standard deviations (SD) for continuous variables and numbers and percentages for categorical variables. As preliminary analysis, we examined the group difference in adherence using t-test for continuous variables and Fisher's exact test for categorical variables. Participants' adherence to HEP and device wear level were also summarized using means and SD. For adherence, average percent HEP adherence (the percentage of prescribed repetitions completed each day averaged over the total duration of participation) was used to represent the person's mean adherence level that is not influenced by dropouts. We also compared individuals' HEP adherence level with device wear level using paired t-test. Similarly, average percent device wear was used for this summary.

For the primary analysis, the SIS change from baseline at each week was the dependent variable. A linear mixed model with group, adherence to HEP, and time (week) along with their interaction as independent variables was performed. Adherence to HEP was quantified as the cumulative number of repetitions completed by each week. To account for within subject repeated measures, a subject level random intercept term was included. Other structures for the within subject correlation were also examined. PROC GLIMMIX in SAS was used for the analysis. Model diagnostics were used to verify normality and model adequacy. In addition, as secondary analysis to explore factors affecting adherence, we examined Pearson correlations between average percent HEP adherence and baseline motor function and self-efficacy.

## 3. Results

### 3.1. Participants

**Figure 2:**
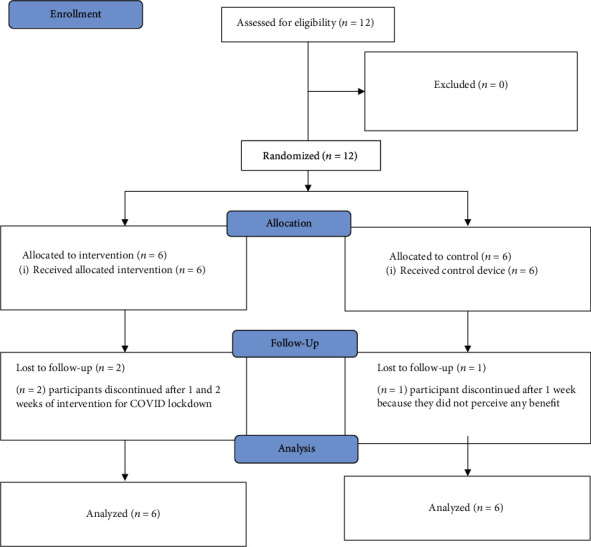
Consort flow diagram.

Twelve chronic stroke survivors with mean ± SD age of 61 ± 10 years participated in the study (see Figure 2 for CONSORT diagram). Baseline characteristics were similar between the two groups (Table 2). During the study period, there were no adverse events reported by participants in the treatment group. However, one participant in the control group reported increased pain, tone, and stiffness with HEP. As for blinding, three participants reported feeling vibration briefly. Two were in the treatment group, and one was in the control group.

### 3.2. HEP Adherence

The average percent adherence to HEP was similar between the two groups (71 ± 39% and 77 ± 40% for the treatment and control group, respectively). However, the average percent adherence to HEP varied across participants, ranging from 7% to 119%. Eight of the 12 participants did not meet the prescribed HEP of 200 repetitions per day, 5 days per week.

### 3.3. Device Wear

Device wear was similar between groups (treatment 108 ± 36%, control 129 ± 34%). Ten participants wore the device as instructed, for at least 8 hours per day. The other two participants, one in each group, still wore the device on average 6 and 9 hours per day on the days that s/he performed HEP. Participants wore the device as instructed more than they adhered to HEP (119 ± 35% for device wear vs. 74 ± 38% for HEP adherence). Since the device was worn for longer durations than HEP, device wear was not included as a covariate in the primary analysis for the hand function outcome below.

### 3.4. Hand Function Outcome

**Figure 3:**
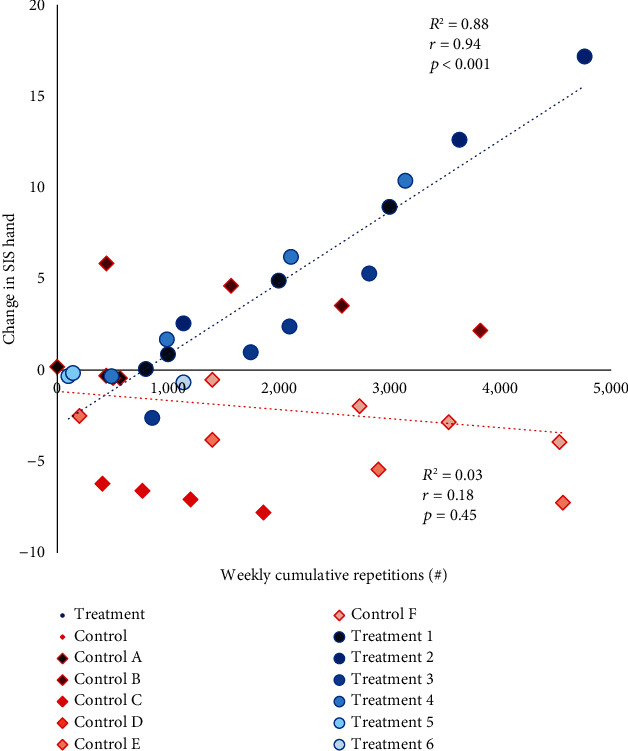
Relationship between cumulative repetitions for HEP vs. fitted model change in Stroke Impact Scale (SIS) Hand domain from baseline, at each of 4 weeks. Treatment group participants are represented by blue circles, and control group participants are represented by orange diamonds. Individual participants are numbered 1-6 for the treatment group (a–f) and for the control group and colored with a different gradient. There are multiple data points per participant because each participant was assessed multiple times.

SIS Hand domain scores were comparable between the two groups at baseline (mean ± SD for the treatment group = 69.2 ± 33.4, control group = 61.7 ± 35.0; *p* = 0.71). The primary linear mixed model analysis showed that the change in SIS Hand was affected by the HEP adherence differently for the two groups (*p* = 0.01 for the interaction effect). Specifically, greater weekly cumulative repetitions in HEP resulted in greater improvement of SIS Hand in the treatment group (Pearson *r* = 0.94; *R*^2^ = 0.88; *p* < .001), while there was no improvement observed in the control group (Pearson *r* = −0.18; *R*^2^ = 0.03; *p* = .45) (Figure 3). Final SIS Hand domain scores were 76.7 ± 28.9 for the treatment group and 52.9 ± 36.4 for the control group.

### 3.5. Factors for Adherence

**Figure 4:**
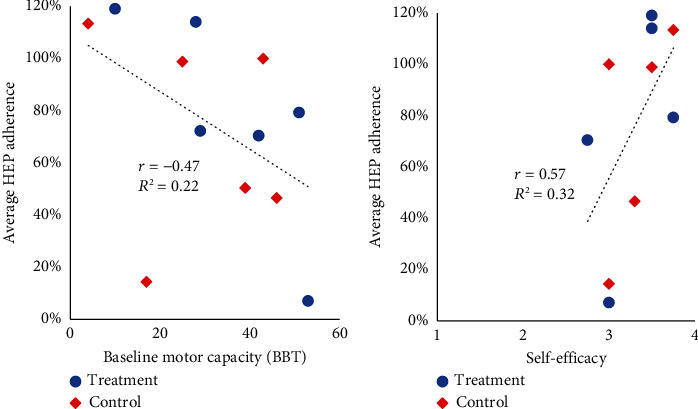
Relationship between baseline motor capacity measured by the Box and Block Test (BBT) score and the average percent HEP adherence (a). Relationship between self-efficacy and average percent HEP adherence (b). The treatment group participants are represented by blue circles and control group participants are represented by orange diamonds.

While adherence was similar between groups, HEP adherence was greater for participants with lower motor capacity measured by BBT (Figure 4(a)). In addition, HEP adherence was greater for those with greater self-efficacy (Figure 4(b)).

## 4. Discussion

The aim of this pilot study was to determine the effects of adherence to HEP combined with the use of TheraBracelet on hand function for stroke survivors. There was a statistically significant interaction between groups and HEP adherence for hand function measured by the SIS Hand. Greater HEP adherence combined with TheraBracelet treatment resulted in increased perceived hand function (Figure 3). This interaction effect was statistically significant in the weekly analysis. In a posthoc analysis examining only the baseline to post changes, similar trends were observed. This trend supports the need for a future study to investigate the effect among a larger sample and over a longer intervention duration or greater dosage as discussed below.

Findings from this study are in concordance with findings from previous studies supporting the use of subsensory vibration to improve upper extremity motor recovery after stroke [21, 23, 36, 37]. This study expands upon previous knowledge [25] by demonstrating that not only wearing a device delivering TheraBracelet stimulation daily is feasible for stroke survivors [24, 25] but also the addition of HEP to TheraBracelet is an important component of improving hand function. Furthermore, while the previous research showed efficacy of TheraBracelet with laboratory-based task practice therapy sessions [23], this study suggests that an independently performed, home-based exercise program combined with TheraBracelet could improve the hand function. Results from the present study encourage a larger study adequately powered to determine the efficacy of TheraBracelet combined with a HEP to improve hand function.

The mean change in hand function measured by SIS Hand domain did not exceed the minimal detectable change (MDC = 17.8) or minimal clinically important difference (MCID = 25.9) [69]. Previous research showed that lab-based therapy with TheraBracelet showed progressive improvement in hand function week by week over the 6-week period, resulting in clinically meaningful improvement in the SIS Hand and Activities of Daily Living domains [23]. With the overall literature supporting greater treatment dose leading to greater improvement [5–9], a longer intervention duration, greater dosage, or higher adherence may be necessary to create a change that is clinically significant.

Previous research suggests adherence is affected by psychosocial factors, including self-efficacy, as was found in this study [62, 70]. Specifically, interviews showed stroke survivors perceived self-efficacy as an important factor for participating in daily physical activity [70], and a prospective study of older adults with recent stroke demonstrated that those with high self-efficacy had greater improvements in balance than those with low self-efficacy [62]. These findings indicate that self-efficacy influences adherence to activity, and the current study supports the importance of self-efficacy in adherence to UE HEP among stroke survivors. As a result, self-efficacy and other psychosocial factors should be investigated to include in the development of behavioral interventions to increase HEP adherence and improve motor recovery after stroke [71, 72].

In this study, we found that lower motor capacity at baseline, measured by the BBT, was associated with greater HEP adherence. Previous literature showed that improving health conditions and functional abilities are strong motivators to exercise [73, 74]. Individuals with lower motor capacity may be more motivated to adhere to HEP because of the desire to improve. Clinicians helping patients increase adherence to HEP may emphasize the importance of HEP adherence for patients to improve functional recovery.

### 4.1. Limitations

A major limitation of this study was the necessity of using self-reported measures for both HEP adherence and hand function. Measuring HEP adherence by participant self-report is known to introduce inaccuracy [14–16]. Accuracy in the measurement of adherence could be improved through the development of technology to objectively measure the UE activity of patients during their activities of daily living [75]. Additionally, hand function was measured by the self-reported SIS Hand domain instead of in-person objective assessments due to COVID-19 lockdown. While the SIS Hand domain provides insight into participants' perceived hand function, objective physical performance outcomes would provide more objective and clinically meaningful data. Additionally, the sample size for this study was small. Future studies will require a larger sample to adequately determine effects in objective functional performance measures.

## 5. Conclusion

This pilot study suggests that adherence to self-directed therapy at home combined with subsensory TheraBracelet stimulation may improve upper extremity recovery outcomes in stroke survivors. The clinical implication of these findings is increased need to effectively promote adherence to prescribed HEP. Additionally, more research is indicated to investigate the effectiveness of TheraBracelet in facilitating recovery among a larger sample of stroke survivors.

## Figures and Tables

**Table 1:** Example Home Exercise Program Tasks with Options to Grade.

Task	Possible adjustments	
	Make easier	Make harder
Description: touch iPad 1 repetition = touch screen 1 time (i.e. open app)	Touch larger icon.	Turn on iPad.
		Play game using left hand.
		Type using left hand.
		Touch smaller icon.

Description: wash dishes 1 repetition = scrub dish 1 time	Hold dish with left hand and scrub with right hand.	Hold dish with right hand and scrub with left.
	Wash lighter/smaller dishes.	Wash larger/heavier dishes.
	Scrub with large sponge.	Scrub with small sponge or rag.

Description: thread needle 1 repetition = thread needle 1 time	Use larger thread and needle.	Use regular sized thread/needle.
	Hold needle with left hand and thread with right hand.	Hold needle with right hand and thread with left hand.

Description: cut food 1 repetition = cut one slice	Cut softer foods (banana).	Cut tougher foods (meat).
	Use left hand to hold fork in food; cut with right hand.	Cut with knife in left hand.

**Table 2:** Baseline characteristics.

	Treatment group (*n* = 6)	Control group (*n* = 6)	*p* value
Sex, *n* (%) male/female	4 (67%)/2 (33%)	2 (33%)/4 (67%)	0.6
Age in years, mean ± SD	60 ± 11	61 ± 9	0.9
Month post stroke, mean ± SD	62 ± 43	51 ± 33	0.6
Type of stroke, *n* (%) ischemic/hemorrhagic	5 (83%)/1 (17%)	4 (67%)/2 (33%)	1.0
Upper extremity Fugl-Meyer, mean ± SD	49 ± 13	48 ± 10	0.9
Box and block test score, mean ± SD	36 ± 17	29 ± 17	0.5
Self-efficacy score, mean ± SD	3.3 ± 0.4	3.3 ± 0.3	1.0

**Table 3:** Self-efficacy questionnaire.

I am the person who is responsible for taking initiatives in my recovery.	Disagree strongly (1)	Disagree (2)	Agree (3)	Agree strongly (4)
Taking an active role in my therapy is important for my health.	Disagree strongly (1)	Disagree (2)	Agree (3)	Agree strongly (4)
I know what treatments are available for my upper limb impairment.	Disagree strongly (1)	Disagree (2)	Agree (3)	Agree strongly (4)
I am confident that I can follow through therapy activities at home.	Disagree strongly (1)	Disagree (2)	Agree (3)	Agree strongly (4)

## Data Availability

Data is available upon request to the corresponding author, Dr. Gabrielle Scronce, at scronce@musc.edu.

## Appendix

The following is a 4-item, self-reported measure of self-efficacy for behavioral activation, which is defined as engagement in health and self-care activities that result in better health and functioning [65–67]. This 4-item measure was loosely adapted from the Patient Activation Measure. The 22-item Patient Activation Measure is a valid and reliable measure of activation, but many items were not relevant to this study. Consequently, we developed the following questionnaire (Table 3) to more accurately reflect the concepts of activation needed for successful participation in a home-based UE rehabilitation therapy program. Participants were asked to rate their agreement with each statement on a scale of 1 (disagree strongly) to 4 (agree strongly) [67].
